# Early Aging in Chernobyl Clean-Up Workers: Long-Term Study

**DOI:** 10.1155/2015/948473

**Published:** 2015-01-27

**Authors:** V. Krasnov, V. Kryukov, E. Samedova, I. Emelianova, I. Ryzhova

**Affiliations:** ^1^Moscow Research Institute of Psychiatry, Poteshnaya Street 3, Moscow 107076, Russia; ^2^Russian National Research Medical University, Moscow 117997, Russia

## Abstract

This paper represents data of long-term open prospective study. 312 male clean-up workers, who participated in elimination of the Chernobyl disaster consequences in 1986-87, were observed and examined in Moscow Research Institute of Psychiatry. The average age of patients was 57,0 ± 6,8 years. All patients were diagnosed with psychoorganic syndrome, caused by combination of different factors, which led to early cerebrovascular pathology, which was confirmed by clinical, neuropsychological, and instrumental examination. Anamnesis and the level of social adaptation were also assayed. Clinical estimation was done with the use of specially developed Clinical Psychopathological Chart. All the symptoms were divided into 4 groups (asthenic, psychovegetative, dysthymic, and cognitive symptom-complexes). No pronounced signs of dementia were observed. The control group included 44 clean-up workers without mental disorders. Predomination of various exogenous factors before and after accident was noted. Therapy included different vasotropic remedies, as well as family therapy, art therapy, and cognitive training. The possibilities of the reverse development of symptoms were statistically proved. The results allow making a conclusion that these disorders could not be explained either by radiation effects or by PTSD but connected with cerebrovascular pathology.

## 1. Introduction

The Chernobyl disaster that happened in 1986 remains a significant medical and social problem [1–4]. Despite the fact that most of the clean-up workers, involved in the elimination of Chernobyl disaster consequences, were exposed to low doses of radiation (usually not exceeding 0,25 Gy, registered by personal dosimeters), at the early stage it was revealed that they have different polymorphic symptoms with combination of asthenic, psychovegetative, and subaffective disorders, at first with mild, neurodynamic, and reversible cognitive disorders, developing later into a psychoorganic syndrome, based on the early cerebrovascular pathology, as well as neuroendocrine, neurological, immune, and psychosomatic disturbances. The polymorphism of the disorders, detected among these patients, and also the fact that they did not have any form of radiation disease made it difficult to interpret them in one strict way and showed the probable multifactorial genesis of these disorders. Among them are extreme conditions of work, sleep irregularity, radioactive fallouts, thrown during the first month of catastrophe, combustion products, toxic elements in decontamination agents and burning of different materials, and also exhalation of boron and lead (sand, containing lead and boron, was strewed into destroyed reactor). Psychological factors may also have been involved, but they seemed to have secondary influence, as PTSD in typical form was not observed in this cohort of patients. The symptomatology did not meet any form of radiation disease. From the beginning we had doubts in the very often categorization of polymorphous clinical symptomatology in clean-up workers as the specific of PTSD, with a projection of fear to “invisible threat.” That interpretation of the nature of the clinical conditions in clean-up workers is usually proposed by psychologists [4–6]. It was quite obvious, as clean-up workers did not have any flashbacks, which are the core symptoms of PTSD, or nightmares, that they did not develop distinct depressive symptoms (just mild subdepressive fluctuations) and were rather socialized at that period of time.

Our clinical experience and traditional approaches allowed us to identify the states, observed in this cohort, as psychoorganic syndrome, the term which is almost forgotten but shows most accurately the entire spectrum of the disorders revealed. The notion of psychoorganic syndrome was coined by Bleuler in 1916 and 1923 [7] and developed by Walther-Büel [8]. It includes the memory and attention impairments (up to difficulties in cognition), combined with affective and vegetative instability.

Psychoorganic syndrome was presented by mild, nondement forms and was mostly caused by an early atherosclerosis and brain vessels angiopathy, caused by hypertension. The structure of this syndrome is presented by 4 main symptom-complexes, asthenic, psychovegetative, dysthymic, and cognitive, and the syndrome develops from early, asthenic or psychovegetative variants, which includes severe headaches, tiredness, irritability, blood pressure fluctuations, and sleep disorders, to the more severe dysthymic variant, and then turns into predement, cognitive deficit form with decreasing intellectual productivity, which does not seem to reverse after the treatment.

Moscow Research Institute of Psychiatry has been involved in health investigation and treatment of Chernobyl clean-up workers. From 1990 till nowadays these patients are treated in our clinic every year (some do twice a year) and receive vasotropic and cerebroprotective therapy, as well as psychosocial therapy, group therapy, and cognitive training. During the course of treatment they are examined by different specialists: therapists, neurologists, ophthalmologists, and endocrinologists. From 1990 to 2014 this cohort of patients, observed and treated at least during 5 consecutive years, achieved 663 patients (the whole number of investigated clean-workers counted 1750). Additionally we often provide consultative assistance to the offsprings of the Chernobyl clean-up workers as part of the family therapy. During the last 5 years the cohort has changed, as several patients died mostly from stroke or heart infarction, cancers or did not enter the cohort by other reasons, and the new patients, who never received regular vasotropic and/or cerebroprotective therapy, have joined. For analysis in this paper we limited the sample to 312 patients which came to our clinic for repeated hospital treatment in the last 5 years. Comparing results of the therapy of patients of this sample with previous therapy results would be incorrect, mostly due to the differences in age. In this paper the results of long-term 5-year prospective study are presented.

## 2. Material and Methods

312 male rescue workers were observed in Moscow Research Institute of Psychiatry from 2009 to 2014. The age of patients varied from 45 to 70 years (the average age 57,0 ± 6,8). The radiation doses usually did not exceed 0,25 Gy. All of the patients were diagnosed with psychoorganic syndrome, confirmed by clinical, neuropsychological, and instrumental examination. Anamnesis and the level of social adaptation were also estimated. Clinical estimation was done by the use of specially developed Clinical Psychopathological Chart [1, 2], Hamilton Depression Scale (HDRS-17), and Hamilton Anxiety Scale (HARS). Neuropsychological part included tests on working memory, attention, and abstract thinking. Instrumental examination included brain MRI and Doppler ultrasound. We did only routine blood tests. Cytological blood tests of clean-up workers were done in another research centre. In some cases they revealed certain aberrations. The assessment of cognitive training effectiveness, comparing before and after treatment, was carried out. Pharmacological treatment was based on vasotropic and cerebroprotective remedies, used intravenously. Statistical data analysis was done with the use of nonparametric tests.

## 3. Results

All the patients were diagnosed with mild, nondement form of psychoorganic syndrome, which included 4 main groups of symptoms: asthenic (permanent headaches, tiredness, irritability, hypothymic reactions, and hyperesthesia), psychovegetative (sleep disorders, permanent or paroxysmal vegetative disorders, paresthesia, algia and other somatoform disorders, low-grade fever, blood pressure fluctuations, etc.), dysthymic (mild dysthymic-dysphoric or anxious-dysphoric depressions usually with inverted circadian status rhythmicity and hyperesthesia), and cognitive (working memory impairments, decreased productivity, and lack of abstract thinking). Nondement cognitive disorders fall under the concept of “disexecutive syndrome,” which includes lack of voluntary attention and initiation, poor planning and outcome control, lack of cognitive flexibility with poor ability to suppress interfering influences, and working memory impairment [9, 10]. In neuropsychological concept all cognitive impairments, observed in this cohort, can be combined in 3 syndromes: diencephalic, diencephalic-right-hemispheric, and diencephalic-frontal (by conception of [11]).

**Figure 1:**
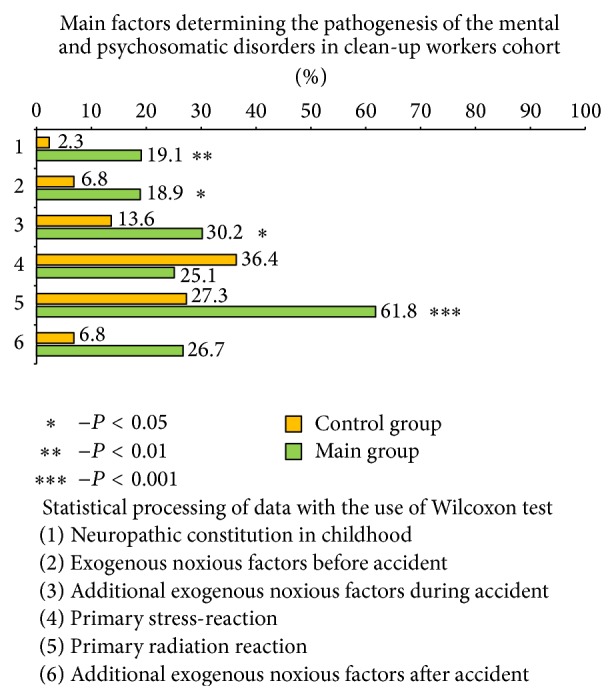


Before assay results it is important to estimate factors, which determined the development of psychoorganic syndrome in clean-up workers cohort, for better understanding of its triggers and pathogenesis. Those clean-up workers, who did not reveal any mental disorders, were the control group (44 patients). The data is presented in Figure 1.

The most significant factor, which influenced the pathogenesis of psychoorganic syndrome, was the primary radiation reaction (statistically significant difference with control group is shown). It included dyspeptic disorders, nausea, tiredness, dizziness, taste and olfactory disorders, and body temperature fluctuations. Primary radiation reaction, with the other factors, could be a trigger for further development of early vascular pathology and further psychoorganic disorders, associated with it.

Neuropathic constitution in childhood, which includes enuresis, ticks, and stammering, was also involved in pathogenesis. These features were mentioned in 19,1% of patients in main group and 2,3% in control group (*P* < 0,01). Neuropathic constitution is not pathology, but it can reduce compensatory abilities and make an unfavourable background for further disorders.

Various exogenous factors before accident, including mild brain injuries and inflammations, were also analysed. As it is mentioned in Figure 1, these factors were observed to be much more frequent in main group than in control group (*P* < 0,05). The more significant difference is shown in additional exogenous factors during accident. They included harmful toxic elements in decontamination substances, exhalation of boron and lead, combustion products, and extreme conditions of work. Statistical data show significant differences between main and control group (*P* < 0,05).

The clean-up workers were exposed to different exogenous factors after an accident too, such as brain trauma, inflammatory diseases, and alcohol abuse, which have also influenced the conditions. The difference between main and control group was statistically significant (*P* < 0,01); the main group shows much more exogenous factors observed than the control group. There were primary stress-reactions during the accident, observed in 25,1% of patients of main group and 36,4% of patients of control group, with no significant difference between them (*P* > 0,05).

These findings indicate predominantly multiple etiology and complex pathogenesis of these disorders, as evidenced by its polymorphism. Polymorphic disorders, as well as somatic and neurological diseases, diagnosed in these patients, require special therapeutic approaches.

The comparative therapeutic 5-year study was carried out with the use of 4 vasotropic remedies and cerebroprotectors: cerebrolysin, vinpocetine (cavinton), actovegin, and picamilon (Table 1).

**Figure 2:**
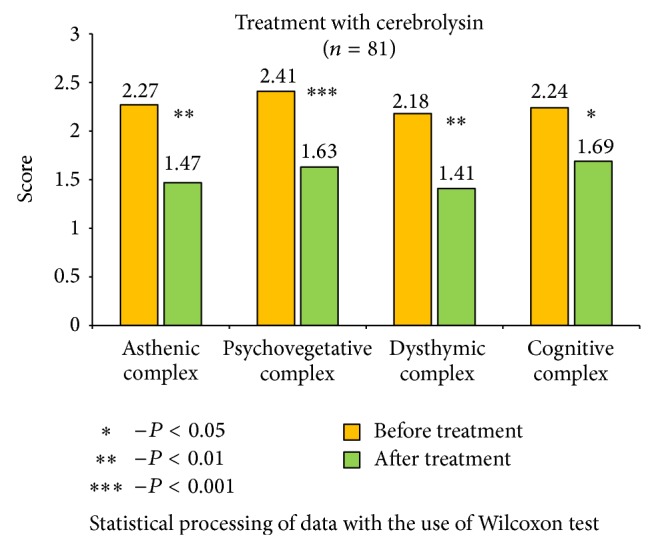


**Figure 3:**
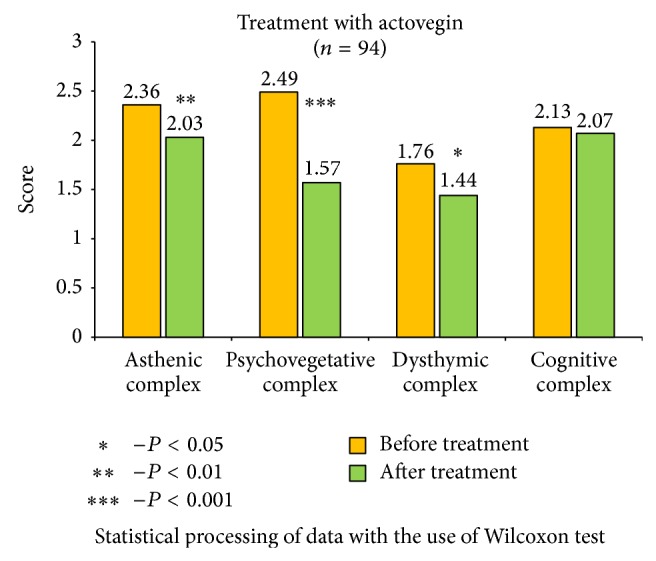


**Figure 4:**
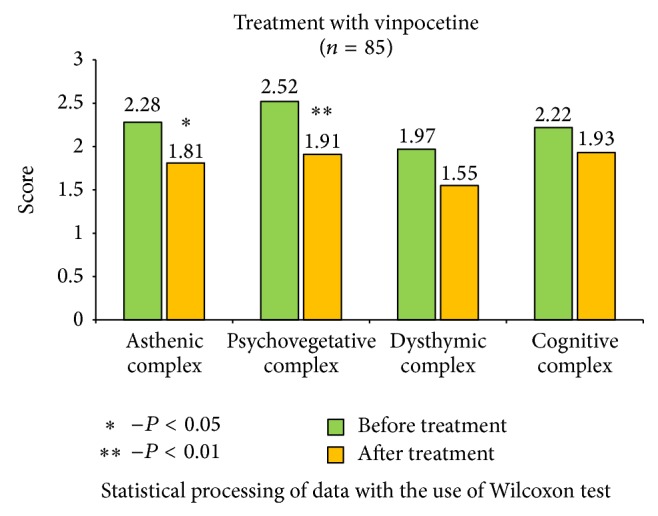


**Figure 5:**
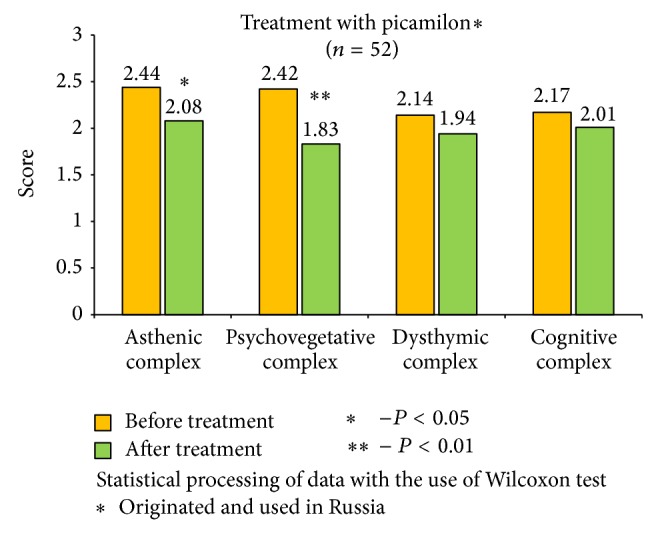


The received data show significant improvements in the health status of patients, with the most notable results in cerebrolysin group (Figure 2). It is important to mention that cerebrolysin had positive impact not only on asthenic and psychovegetative symptom-complex, but on the level of dysthymic disorders (*P* < 0,01) and cognitive functions as well (*P* < 0,05). Other remedies also reduced asthenic, psychovegetative, and dysthymic disorders, and the most effective results were identified in actovegin group (Figure 3); significant reduction of asthenic and psychovegetative disorders was mentioned in vinpocetine and picamilon group, with no statistically significant improvements in dysthymic and cognitive complex (Figures 4 and 5).

**Figure 6:**
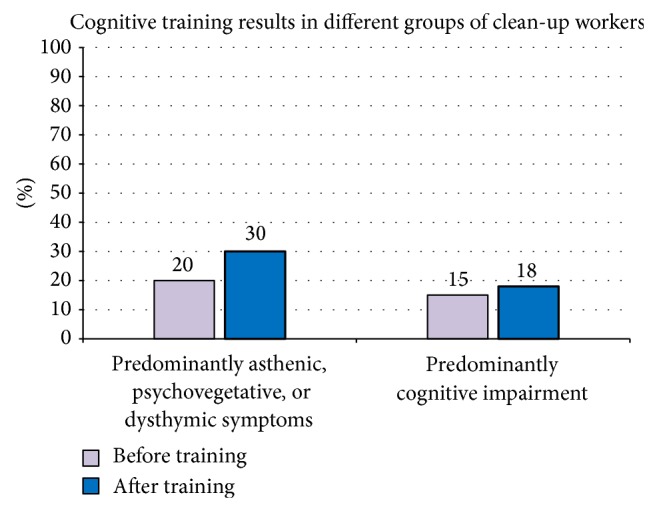


The therapy of this cohort of patients also included psychosocial work, art therapy, family therapy, and cognitive training. The analysis of results is represented in 2 main groups: patients with predominant asthenic, psychovegetative, or dysthymic disorders and patients with predominant cognitive impairment (Figure 6). Both groups showed reduction of cognitive disorders after the training course, but patients with more severe cognitive impairment improved their cognitive skills markedly less.

The verification of cerebrovascular pathology and assessment of the brain damage level was carried out with the use of brain MRI and Doppler ultrasound. It is important to mention severe, predominantly diffuse changes in brain in all the patients of this cohort, with the high frequency of leukoaraiosis, despite relatively low expression of these changes. The level of leukoaraiosis correlated with the severity of the psychopathological disorders, estimated by the clinical chart (*r* = 0,41, *P* < 0,05).

The data analysis of variance showed a statistically significant dependence of internal hydrocephalus from prevailing clinical symptoms (*F* = 2.75; *P* < 0,05).

It should be also noted that the morphology of congenital disorders of the brain acquired anatomical features of the brain tissue and changes in the pituitary gland are rare finds and occur in isolated cases.

The evaluation of Doppler ultrasound results showed that the level of blood flow in cerebrovascular system was characterized by rather frequent occurrence (80%) and mild to moderate circulatory disorders. There were no severe pathologies, but presented disorders were characterized by damage in both carotid and vertebral-basilar arteries and venous portion of the vascular system.

Noteworthy is prevalence of impaired blood flow rate in the system of the carotid arteries, especially in its extracranial branches (85%), as well as the involvement of the arterial system in the pathological process at its intracranial level (64%), indicating the active involvement of compensatory mechanisms in the regulation of brain blood supply. In 87% of cases difficulty in venous outflow was noted.

In our earlier papers the diffuse brain lesions were detected by the data of SPECT (single photon emission tomography) [1, 2]. Our data regarding early vascular pathology coincides with results of studies by neurologists [12–15].

## 4. Conclusions

Polymorphic disorders in clean-up workers cohort, which fit into the concept of psychoorganic syndrome, are based on multifactorial etiology and specific pathogenesis, with the influence of biological, psychological, and social factors. The complex of these factors leads to early cerebrovascular pathology that manifests a variety of symptom-complexes, with the core symptom-complex of cognitive impairment. Regular treatment, which includes stationary annual courses with vasotropic and cerebroprotective remedies, as well as psychosocial work, including family therapy, group therapy, and cognitive training, can significantly reduce symptoms of disease and improve volitional resources, which is the most reasonable approach, as it contributes to personality preservation and improves social adaptation of patients.

## Figures and Tables

**Table 1:** 

	Remedy	Daily dose	Type of treatment	Number of patients
1	Cerebrolysin	10–20 mL	Intravenously, #20	81
2	Vinpocetine	10 mg	Intravenously, #20	85
3	Actovegin	80–160 mg	Intravenously, #20	94
4	Picamilon	100–200 mg	Intravenously, #20	52

## References

[1] Krasnov V. N., Petrenko V. E., Voitsekh V. F. (1993). Mental disturbances in liquidators of the aftermath of the Chernobyl disaster. 2. Clinical-pathogenetic and pathoplastic relationships. *Social and Clinical Psychiatry*.

[2] Krasnov V. N., Yurkin M. M., Voitsekh V. F. (1993). Mental disturbances in liquidators of the aftermath of the Chernobyl disaster. *Social and Clinical Psychiatry*.

[3] Krasnov V. N. (2004). Psychopathological consequences of the Chernobyl disaster as a subject of ecological psychiatry. *Italian Journal of Psychiatry and Behavioural Sciences*.

[4] Speckhard A., Berkowitz N. (2005). Psycho-social and physical outcomes of technological disaster; information as a traumatic stressor. *A Chernobyl Reader*.

[5] Tarabrina N. V., Lazebnaya E., Zelenova M., Lasko N. (1996). Chernobyl clean-up workers' perception of radiation threat. *Radiation Protection Dosimetry*.

[6] Bromet E. J., Havenaar J. M. (2007). Psychological and perceived health effects of the chernobyl disaster: A 20-year review. *Health Physics*.

[7] Bleuler E. (1923). *Lehrbuch der Psychiatrie*.

[8] Walther-Büel H. (1951). *Die Psychiatrie der Hirngeschwülste und die Cerebralen Grundlagen Psychischer Vorgänge*.

[9] Hanna-Pladdy B. (2007). Dysexecutive syndromes in neurologic disease. *Journal of Neurologic Physical Therapy*.

[10] Lezak M. D., Howieson D. B., Bigler E. D., Tranel D. (2013). *Neuropsychological Assessment*.

[11] Luria A. R. (1966). *Higher Cortical Functions in Man*.

[12] Denisuk N. V. (2006). Chronic cerebrovascular disorders within remote period upon radiation exposure in Chernobyl NPP accident clean-up workers. *Ukrainian Medical Journal*.

[13] Antypchuk Y. Y., Loganovsky K. N., Perchuk I. V., Loganovskaja T. K., Chumak S. A., Kreinis G. Y. (2008). Postradiation cognitive disorders. *International Journal of Psychophysiology*.

[14] Kholodova N. B., Zhavoronkova L. A. (2011). Changes in the nervous system in chernobyl nuclear power plant accident clean-up workers. *Neuroscience and Behavioral Physiology*.

[15] Loganovsky K. N., Zdanevich N. A. (2013). Cerebral basis of posttraumatic stress disorder following the Chernobyl disaster. *CNS Spectrums*.

